# Educational Value of a One-Week Mandatory Anesthesiology Clerkship: A Cross-Sectional Comparison of Medical Students’ Perceptions, Learning Preferences, and Career Interest

**DOI:** 10.7759/cureus.106166

**Published:** 2026-03-30

**Authors:** Amal F Farouk, Yazan Chaiah, Mhd Malek Al Masri, Omar H Barbour, Yazan Almasry, Kenan A Shawwaf, Al-Awwab M Dabaliz

**Affiliations:** 1 Department of Anesthesiology and Perioperative Medicine, University Hospitals Cleveland Medical Center, Cleveland, USA; 2 Department of Anesthesiology, Alfaisal University College of Medicine, Riyadh, SAU; 3 Department of Surgery, University of Illinois Chicago, Chicago, USA; 4 Department of Anesthesiology, University of Illinois Chicago, Chicago, USA

**Keywords:** anesthesiology, career choice, clerkship, clinical exposure, curriculum design, experiential learning, medical education, perioperative medicine, simulation, undergraduate training

## Abstract

Background: One‑week anesthesiology clerkships are common in undergraduate medical curricula, yet their educational yield and influence on career interest remain unclear. Longer rotations have demonstrated improvements in attitudes and specialty consideration, but comparable evidence for short mandatory experiences is limited. This study compared perceptions of anesthesiology among students surveyed before and after a structured one‑week clerkship.

Methods: A cross‑sectional design was used at a single medical school. Two independent cohorts of fifth‑year students completed a validated questionnaire: a pre‑clerkship cohort (n = 58) was surveyed before starting the rotation, and a post‑clerkship cohort (n = 54) was surveyed immediately after the final examination. The survey assessed career preferences, attitudes toward anesthesiology, perceived barriers, and preferred learning modalities. Post‑clerkship respondents additionally evaluated satisfaction and perceived educational gains.

Results: A total of 112 students participated (70% response rate). No statistically significant differences were observed across the 13 perception items after Bonferroni correction (all p > 0.0038; effect sizes r = 0.018-0.125). Post‑clerkship students reported improved anesthesia‑related knowledge (55.6%; 30/54) and improved procedural skills (50.0%; 27/54), particularly in airway management and intravenous access. Only 16.7% (9/54) reported increased interest in anesthesiology as a career. Satisfaction with rotation length was low (13.0%; 7/54). Learning preferences shifted toward hands‑on workshops (42.6% [23/54] post‑clerkship vs. 29.3% [17/58] pre‑clerkship).

Conclusions: Post-clerkship students more frequently reported anesthesia-related knowledge gains (55.6%; 30/54) and improved procedural skills (50.0%; 27/54) than pre-clerkship students, yet no between-group differences in attitudes or career interest were observed (16.7% [9/54] reporting increased interest). This null finding contributes positively to the evidence base on minimum effective exposure thresholds: one week appears sufficient for foundational learning but insufficient to influence specialty consideration. A notable shift toward hands-on workshops (from 29.3% [17/58] to 42.6% [23/54]) offers an actionable design implication. Longer or longitudinally integrated models are recommended to achieve attitudinal and career-related impact.

## Introduction

Anesthesiology plays a central role in perioperative medicine, critical care, and pain management, yet medical students often have a limited understanding of the specialty’s scope and responsibilities. Studies across multiple countries show that students frequently underestimate anesthesiologists’ involvement in perioperative decision-making, patient safety, and critical care, reinforcing persistent misconceptions about the specialty. Adudu found that Nigerian medical students viewed anesthesiologists primarily as technicians rather than perioperative physicians [1], while Adudu et al. documented similarly limited understanding among students in Canada [2]. Nekyon et al. confirmed that these misconceptions extend broadly across sub-Saharan African programs [3], and Nixon et al. reported that even students in well-resourced Canadian institutions lacked understanding of anesthesiology practice [4]. These gaps are important because early perceptions shape specialty consideration and professional identity in ways that are difficult to reverse later in training. Dorsey et al. demonstrated that lifestyle factors and early impressions of a specialty’s prestige are among the strongest predictors of career interest [5]; Gqiba et al. showed that students actively avoid specialties they perceive as less engaging or stressful [6]; and Borges et al. found that intrinsic motivators, including early clinical experiences, disproportionately influence specialty choice among contemporary medical students [7]. In this study, “perceptions” refer specifically to students’ attitudes, beliefs, and assumptions about anesthesiology.

Structured anesthesiology rotations have been shown to improve students’ knowledge, procedural confidence, and appreciation of the specialty’s complexity, particularly when well-designed and sufficiently long. Galway reported that 96% of students completing a four-week elective expressed increased interest in anesthesiology [8], while Khan et al. found that a two-week structured rotation significantly raised the proportion of students ranking anesthesiology among their top career choices [9]. Long et al. similarly reported that early structured exposure increased the likelihood of specialty consideration [10]. Bohrer et al. further documented improvements in procedural confidence following well-designed procedural training sessions [11]. Longer rotations allow students to develop meaningful mentor-mentee relationships with faculty, a condition Sambunjak et al. identified as central to specialty interest formation [12]. However, many medical schools worldwide offer only brief exposure to anesthesiology, often limited to a single week embedded within surgical or emergency medicine clerkships [13]. The educational value and long-term attitudinal impact of these short mandatory rotations remain poorly understood.

This study is grounded in two complementary educational frameworks. Kirkpatrick’s model of training evaluation emphasizes assessing learner reaction, perceived learning, and behavioral intention-domains, particularly relevant to short, time-limited educational interventions [14]. Short clerkships may effectively influence satisfaction and perceived knowledge (Levels 1 and 2) but are less likely to produce changes in specialty interest (Level 3). Kolb’s experiential learning theory further suggests that meaningful learning requires iterative cycles of concrete experience, reflective observation, abstract conceptualization, and active experimentation [15]. A one-week rotation provides limited opportunity for these cycles to unfold, raising questions about whether such brief exposure can meaningfully shape students’ attitudes or correct longstanding misconceptions.

Simulation-based education has become increasingly important in anesthesiology training. Issenberg et al. identified deliberate practice and immediate feedback as the features most strongly associated with effective simulation-based learning [16], while Okuda et al. documented that simulation improves learner confidence and skill acquisition across a range of procedural tasks [17]. McGaghie et al. demonstrated that mastery learning frameworks, in which learners practice until they reach a defined competency threshold, produce particularly durable skill retention [18]. Barsuk et al. showed that simulation-based training directly translates into improved procedural performance in clinical settings [19]. Short rotations that incorporate simulation may therefore provide meaningful educational value despite limited clinical exposure. Additionally, contemporary models of clinical learning emphasize the importance of workplace-based experiences, reflective practice, and supportive learning environments [20,21], all of which may influence the effectiveness of short clerkships.

Despite the prevalence of one-week anesthesiology rotations, few studies have rigorously evaluated their impact using validated instruments and established educational frameworks. To address this gap, the present study compares perceptions, learning preferences, and career interest among medical students surveyed before and after completing a structured one-week anesthesiology clerkship. By situating the findings within established educational theory and the broader anesthesiology education literature, this study provides evidence to guide the design of short clerkships and informs discussions about the minimum exposure necessary to support meaningful learning and career exploration in the perioperative sciences. We hypothesize that the post-exposure group will demonstrate improved perception without showing greater interest in pursuing a career in anesthesiology.

## Materials and methods

Study design and setting

This study used a cross‑sectional, questionnaire‑based design conducted at the College of Medicine, Alfaisal University, in Riyadh, Saudi Arabia. Anesthesiology is delivered as a mandatory one‑week clerkship during the fifth year of a six‑year undergraduate medical curriculum. Data were collected from January 2019 to May 2019. The institutional review board approved the study protocol, and participation was voluntary and anonymous.

A cross‑sectional design was selected because logistical constraints prevented surveying the same students before and after the rotation. Instead, two independent cohorts at the same stage of training were compared. Rotation structure, faculty, clinical sites, and assessment timing were constant between cohorts. This design aligns with Kirkpatrick’s model of training evaluation, which supports assessing learner reaction, perceived learning, and behavioral intention even when long‑term outcomes cannot be measured. It also reflects Kolb’s experiential learning theory, which emphasizes that short rotations may influence immediate learning but offer limited opportunity for iterative experiential cycles.

Structure of the one‑week clerkship

The clerkship followed a structured curriculum integrating didactic, experiential, and simulation‑based learning. The rotation incorporated four one‑hour didactic lectures covering pre‑anesthetic assessment, airway management, intravenous anesthetics and hypnotics, and inhalational anesthetic agents; three days of operating room exposure across general surgery, cardiac anesthesia, and obstetric anesthesia; a half‑day pain clinic experience; one full day in the simulation center emphasizing airway management, epidural and spinal placement, and intravenous access; and small‑group discussions on general versus regional anesthesia and invasive versus non‑invasive monitoring.

This structure was intentionally designed to expose students to multiple stages of Kolb’s learning cycle, concrete experience, reflective observation, abstract conceptualization, and active experimentation, within the constraints of a one‑week rotation. A summary of the one-week anesthesiology clerkship structure can be found in Table 1.

**Table 1:** One-week anesthesiology clerkship structure at College of Medicine, Alfaisal University.

Didactic Lectures (1 hour per session)	Other Teaching Activities
Pre-Anesthetic Assessment; Airway Management; IV anesthetics and hypnotics; Inhalational anesthetic agents	1-day workshop in the simulation center covering different anesthetic techniques (e.g., airway management, epidural and spinal placement, etc.).
	Small group discussion sessions covering the differences between general and regional anesthesia as well as invasive and non-invasive monitoring.
Clinical Time (shadowing an anesthesiologist)	Student prepared presentations based on a pre-assigned list of topics.
1 day in General OR; 1 day in Cardiac OR; 1 day in Labor & Delivery; Half day in pain clinic	Some activities (e.g., BLS and ACLS review) may overlap with the Emergency Medicine curriculum as the anesthesiology clerkship is typically delivered as part of a 4-week combined Emergency Medicine (3 weeks) and Anesthesiology rotation.

Participants

All fifth‑year medical students enrolled during the academic year (n = 160) were eligible. Two independent cohorts were formed based on the timing of survey administration. Group 1 (pre‑clerkship) comprised 58 respondents who completed the survey before starting the rotation, and Group 2 (post‑clerkship) comprised 54 respondents who completed the survey immediately after the final examination.

Students from other academic years were excluded. No identifying information was collected, and participation had no impact on academic standing.

Survey instrument

Data were collected using a questionnaire (see Appendix Figure 1) adapted, with permission, from Galway’s validated instrument originally developed for a four‑week elective [8]. Three adaptations were made: contextual modifications for a mandatory one‑week rotation, the addition of items assessing satisfaction with rotation length, and the addition of items assessing perceived procedural skill improvement.

The survey collected demographic data (gender, GPA), rankings of the top three preferred career choices, a 13‑item Likert scale assessing perceptions of anesthesiology (rated 1 = strongly disagree, 2 = disagree, 3 = neutral, 4 = agree, 5 = strongly agree; higher scores indicate stronger agreement with each stated barrier), preferred learning modalities, and open‑ended feedback. Post‑clerkship respondents additionally completed eight items assessing satisfaction with the clerkship and perceived educational gains, rated on the same five‑point scale. Group‑level Likert scores were summarized as medians with interquartile range (Q1-Q3), and between‑group comparisons used the Mann-Whitney U test.

To address construct validity, the adapted instrument underwent expert review by two anesthesiology faculty members to ensure clarity and relevance. Internal consistency of the 13‑item scale demonstrated acceptable reliability (Cronbach’s α = 0.81). Nevertheless, adapting an instrument originally developed for a different rotation length and educational context introduces unavoidable limitations. Differences in curricular structure, cultural expectations, and the nature of a one‑week mandatory clerkship may influence how students interpret certain items. These contextual factors were considered during expert review, but they remain inherent constraints that may affect construct equivalence.

Data collection procedures

Surveys were administered electronically during scheduled academic sessions. Students were informed that participation was voluntary, anonymous, and unrelated to clerkship evaluation. No incentives were provided.

Statistical analysis

Quantitative analyses were conducted using IBM Corp. Released 2021. IBM SPSS Statistics for Windows, Version 27. Armonk, NY: IBM Corp. Descriptive statistics summarized demographic characteristics and response distributions.

Given the ordinal nature of Likert‑scale data and the independent‑samples design, the Mann-Whitney U test was used to compare pre‑ and post‑rotation responses. Because 13 simultaneous comparisons were performed, a Bonferroni correction was applied to control for Type I error inflation, yielding an adjusted significance threshold of α = 0.0038.

Effect sizes (r) were calculated and interpreted using conventional thresholds (small < 0.30, medium 0.30-0.49, large ≥ 0.50). A post‑hoc power analysis was conducted using G*Power 3.1. With a total sample of 112 participants (58 pre-clerkship, 54 post-clerkship), the study had 0.80 power to detect medium effect sizes (r = 0.30) using a two-tailed Mann-Whitney U test at α = 0.05. The study was adequately powered to detect medium effects but underpowered to detect small effects, which may partially explain the uniformly small effect sizes observed.

Open‑ended responses were analyzed using an inductive content‑analysis approach. Because the dataset consisted of short, single‑sentence comments rather than extended narrative text, formal inter‑rater reliability statistics were not calculated. Instead, a consensus‑based agreement process was used, consistent with recommended practices for small qualitative datasets. Themes were derived inductively and used to contextualize quantitative findings.

## Results

Participant characteristics

A total of 112 fifth‑year medical students completed the survey, yielding an overall response rate of 70%. Group 1 (pre‑clerkship) included 58 students (72.5% response rate), and Group 2 (post‑clerkship) included 54 students (67.5% response rate). Gender distribution was identical across groups, with 52% female respondents in each cohort. Academic performance was comparable: 88% of Group 1 and 87% of Group 2 reported a cumulative GPA above 3.0 on a 4.0 scale.

When asked to list their top three preferred career choices, 6.9% of pre‑clerkship students and 7.4% of post‑clerkship students included anesthesiology. Internal medicine, pediatrics, and general surgery were the most frequently selected specialties across both groups.

Perceptions of anesthesiology before and after the clerkship

Students completed a 13‑item Likert scale (Table 2) assessing perceptions of anesthesiology and factors influencing specialty interest. Median scores for all items were similar between groups, with responses frequently clustering around the neutral category. Using the Mann-Whitney U test with Bonferroni correction (α = 0.0038), none of the 13 items demonstrated statistically significant differences between pre‑ and post‑clerkship groups. Effect sizes were uniformly small (r = 0.018-0.125), and 95% confidence intervals for all effect sizes overlapped zero, indicating an absence of meaningful shifts in attitudes across all measured domains. Overall, the item‑level analysis suggests that the one‑week clerkship did not substantively alter students’ perceptions of anesthesiology.

**Table 2:** Comparison of pre‑ and post‑clerkship Likert item scores (Mann–Whitney U test, Bonferroni-corrected α = 0.0038). Scores rated 1 (strongly disagree) to 5 (strongly agree). Data presented as median (Q1–Q3). Effect sizes: small r < 0.30, medium r = 0.30–0.49, large r ≥ 0.50.

Item	Pre-clerkship (n = 58) Median (Q1–Q3)	Post-clerkship (n = 54) Median (Q1–Q3)	U-test	p-value	Effect Size (r)	Interpretation
Lack of understanding of the role of anesthesiologists	3.0 (2.0–4.0)	2.5 (1.2–4.0)	1776	0.2089	0.116	Small
Minimal exposure to the specialty in medical school	4.0 (3.0–5.0)	4.0 (3.0–5.0)	1420	0.3755	0.080	Small
Responsibilities incompatible with traits and expectations	3.0 (3.0–4.0)	3.0 (3.0–4.0)	1464	0.5327	0.056	Small
Anesthesiology has a limited scope of practice	3.0 (2.0–4.0)	3.0 (2.0–4.0)	1644	0.6441	0.043	Small
Anesthesiology is considered less prestigious	2.0 (1.0–3.0)	3.0 (2.0–4.0)	1347	0.1918	0.120	Small
Anesthesiology is less financially rewarding	2.0 (1.0–3.0)	2.0 (1.0–3.0)	1792	0.1704	0.125	Small
Anesthesiology provides minimal patient contact	4.0 (3.0–4.0)	4.0 (3.0–5.0)	1526	0.8106	0.022	Small
Anesthesiology prevents long-term patient relations	4.0 (3.0–5.0)	4.0 (3.0–4.8)	1599	0.8452	0.018	Small
I dislike the operating room setting	3.0 (1.0–4.0)	2.0 (1.0–4.0)	1650	0.6189	0.046	Small
Lack of opportunities for private practice	3.0 (2.0–3.0)	3.0 (2.0–3.0)	1452	0.4894	0.063	Small
Anesthesiology is not challenging and leads to boredom	3.0 (2.0–3.0)	3.0 (2.0–4.0)	1450	0.4892	0.064	Small
Anesthesiology offers limited fellowship opportunities	3.0 (2.0–3.0)	3.0 (2.2–3.0)	1520	0.7736	0.025	Small
Dependency of the specialty on providers from other specialties	3.0 (3.0–4.0)	3.0 (3.0–4.0)	1692	0.4426	0.069	Small

Educational impact of the one‑week clerkship

Among post‑clerkship respondents (Group 2, n = 54), 55.6% (30/54) reported improved anesthesia‑related knowledge and 50.0% (27/54) reported improved procedural skills, particularly in airway management and intravenous line placement. An additional 22.2% (12/54) reported improved general medical knowledge. Despite these perceived learning benefits, only 25.9% (14/54) felt their expectations for the rotation were met, only 13.0% (7/54) were satisfied with the rotation length, and only 16.7% (9/54) reported increased interest in pursuing anesthesiology as a career. Overall satisfaction with the clerkship experience was reported by 31.5% (17/54) of respondents. These results are summarized in Table 3.

**Table 3:** Post-clerkship educational impact among Group 2 respondents (n = 54). Items are rated 1 (strongly disagree) to 5 (strongly agree); agree/strongly agree = score ≥4.

Post-clerkship item (n = 54)	Agree/Strongly Agree (score ≥4)	Median (Q1–Q3)
Improved anesthesia-related knowledge	30/54 (55.6%)	4.0 (3.0–4.0)
Improved procedural skills (airway, IV access)	27/54 (50.0%)	3.5 (2.0–4.0)
Improved general medical knowledge	12/54 (22.2%)	3.0 (2.0–3.0)
Expectations for the rotation were met	14/54 (25.9%)	2.0 (2.0–3.8)
Increased interest in anesthesiology career	9/54 (16.7%)	2.0 (1.2–3.0)
Satisfied with rotation length	7/54 (13.0%)	2.0 (1.0–3.0)
Overall satisfied with clerkship experience	17/54 (31.5%)	3.0 (2.0–4.0)

These findings suggest that while the rotation enhanced foundational understanding and procedural comfort, it did not substantially influence specialty consideration.

Preferred learning modalities

Learning preferences differed markedly between the two groups. Among pre‑clerkship students (Group 1, n = 58), 41.4% (24/58) preferred shadowing an anesthesiologist for a minimum of one week, and 29.3% (17/58) preferred hands‑on workshops, with 12.1% (7/58) each preferring didactic lectures or small‑group discussions. Among post‑clerkship students (Group 2, n = 54), 42.6% (23/54) preferred hands‑on workshops, 31.5% (17/54) preferred shadowing, 13.0% (7/54) preferred small‑group discussions, and only 9.3% (5/54) preferred didactic lectures. These preferences are compared in Table 4.

**Table 4:** Preferred learning modalities before (Group 1) and after (Group 2) the clerkship.

Learning Modality	Group 1 Pre-clerkship (n = 58)	Group 2 Post-clerkship (n = 54)
Hands-on workshop	17 (29.3%)	23 (42.6%)
Shadowing anesthesiologists (≥1 week)	24 (41.4%)	17 (31.5%)
Small-group discussions	7 (12.1%)	7 (13.0%)
Didactic lectures	7 (12.1%)	5 (9.3%)
Not interested / Other	3 (5.2%)	2 (3.7%)

This shift suggests that experiential and simulation‑based learning were perceived as more valuable after students experienced them firsthand.

Qualitative feedback

Open‑ended responses from Group 2 (n = 54) identified three recurring themes. First, multiple students recommended extending the rotation beyond one week, citing insufficient time to develop meaningful familiarity with the specialty. Second, students requested broader subspecialty exposure, particularly access to preoperative assessment clinics. Third, several students suggested embedding anesthesiology more deeply within the surgical clerkship to enhance continuity and contextual relevance.

Only one student explicitly stated that the rotation length was adequate.

## Discussion

This study compared perceptions, learning preferences, and career interest between medical students surveyed before and after completing a structured one‑week mandatory anesthesiology clerkship. Post-clerkship students more frequently reported perceived knowledge gains and procedural confidence than their pre-clerkship counterparts, yet no statistically significant between-group differences were observed in attitudes toward anesthesiology or interest in pursuing the specialty. These findings are consistent with prior work demonstrating that brief rotations primarily influence Kirkpatrick Level 1 (reaction) and Level 2 (learning) outcomes, whereas shifts in specialty interest, analogous to Level 3 (behavioral intention), typically require longer or more immersive experiences [14,22].

Interpretation in the context of existing literature

The absence of significant attitudinal change contrasts with studies of longer anesthesiology rotations. Galway reported substantial increases in student interest following a four‑week elective [8], and Khan et al. observed similar gains after a two‑week structured rotation [9]. It is important to note that both Galway and Khan employed pre‑post within‑subject designs, which provide stronger causal inference regarding attitudinal change. In contrast, the present study used a cross‑sectional between‑groups design, which limits direct comparability and may partially explain the absence of measurable shifts in perceptions.

Long et al. reported that early structured exposure increased the likelihood of considering anesthesiology as a career [10]. These findings suggest that timing and duration are critical determinants of whether exposure translates into attitudinal or career-related shifts. Longer rotations allow students to observe a wider range of perioperative responsibilities and engage in repeated experiential learning cycles-conditions that align more closely with Kolb’s experiential learning theory [15,20,21].

In contrast, one‑week clerkships, while common globally, may not provide sufficient time for students to progress beyond initial exposure. The persistence of these misconceptions across different educational systems underscores the challenge. Adudu and Adudu et al. documented them in Nigerian and Canadian programs, respectively [1,2]; Nekyon et al. reported poor perception of the specialty across different African countries [3]; and Nixon et al. confirmed that even structured Canadian clerkships left some gaps in students’ understanding of the anesthesiologist’s perioperative role [4]. Correcting deeply ingrained misconceptions may require more sustained engagement than a single week can offer.

Educational value of short rotations

Despite the absence of attitudinal change, post-clerkship students reported meaningful educational gains. More than half of post‑clerkship students reported improved anesthesia‑related knowledge and procedural confidence, particularly in airway management and intravenous access. These gains are consistent with evidence that simulation‑based education enhances procedural competence, learner engagement, and confidence in high‑stakes clinical skills. Issenberg et al. established that deliberate practice with feedback is the core mechanism of effective simulation [16], while Barsuk et al. demonstrated that simulation-acquired skills transfer directly to improved clinical performance, a finding particularly relevant to the procedural gains (airway management and intravenous access) reported here [19]. McGaghie et al. further showed that mastery-based simulation produces retention superior to time-limited exposure alone [18]. The shift in preferred learning modalities, from shadowing to hands‑on workshops, further reinforces the value of experiential and simulation‑based approaches in anesthesiology education.

Short rotations may therefore serve an important introductory function: providing foundational exposure, correcting basic misconceptions, and preparing students for more advanced perioperative learning later in training [13].

Why attitudes and career interests did not change

The unchanged proportion of students ranking anesthesiology among their top career choices is consistent with broader literature on specialty selection. Career interest is shaped by multiple factors, including perceived lifestyle, prestige, mentorship, and long‑term patient relationships, that are unlikely to shift during a brief rotation. Dorsey et al. showed that controllable lifestyle and perceived prestige are among the strongest drivers of specialty selection, factors shaped over years rather than days [5]. Gqiba et al. found that students who perceived a specialty as less engaging or stressful actively avoided it regardless of brief exposure [6], while Borges et al. demonstrated that intrinsic motivators established early in training are more influential than short-term experiential encounters [7]. Sambunjak et al. further showed that mentorship relationships, which develop over weeks to months, not days, are one of the most reliable predictors of specialty choice [12]. Additionally, anesthesiology is often underrepresented in preclinical curricula, meaning students may enter the clerkship with limited baseline knowledge or strong pre‑existing preferences.

The uniformly small effect sizes across all perception items suggest that students’ attitudes were relatively stable and not easily influenced by short‑term exposure. This pattern reinforces the idea that attitudinal change requires time, repeated experiential cycles, and opportunities for reflection, core components of Kolb’s model that are difficult to achieve in a one‑week format [15,21].

Comparison to longer and longitudinal models

Evidence from other specialties suggests that longitudinal or repeated exposures are more effective than brief rotations in shaping attitudes and career interest. Longitudinal integrated clerkships have been shown to enhance specialty understanding, strengthen professional identity formation, and improve continuity of learning [20]. In anesthesiology, early exposure has been associated with improved perceptions of the specialty’s scope and increased likelihood of considering it as a career [10].

These findings suggest that while one‑week clerkships provide valuable introductory exposure, they may represent the minimum threshold for meaningful learning rather than the optimal structure for influencing specialty choice. This null career-interest finding is a positive contribution to the evidence base: it establishes an empirically grounded lower bound on the exposure duration required to influence specialty consideration, information that is directly usable by curriculum designers weighing time-allocation decisions. Prior literature documented what longer rotations can achieve; the present study documents what a rigorously structured one-week rotation cannot, a necessary data point for setting realistic expectations and designing appropriate interventions.

Strengths and limitations

This study has several strengths, including the use of a validated instrument, integration of established educational frameworks, transparent statistical reporting with Bonferroni correction, and mixed‑methods analysis. However, several limitations warrant consideration. The cross‑sectional design prevents assessment of within‑student change and introduces the possibility of cohort differences. All outcomes were self‑reported, and no objective assessments of knowledge or skill acquisition were included. The one‑week duration inherently limits the depth of exposure and may not generalize to longer or differently structured rotations. Although the survey was adapted from a validated instrument, modifications for the local context may have influenced construct validity. The study was conducted at a single institution, which may limit external generalizability. Finally, although gender and GPA distributions were comparable between groups, unmeasured cohort differences may still have influenced responses.

Transferability and implications for future research

The transferability of these findings to other curricular models warrants consideration. In four‑year U.S. medical programs, anesthesiology exposure is often elective and may occur later in training, whereas many European and Middle Eastern programs incorporate earlier and more structured perioperative teaching. The limited impact observed in this one‑week rotation may therefore reflect broader international variability in baseline exposure, curricular sequencing, and institutional expectations. Future studies across diverse educational systems are needed to determine whether similar patterns emerge in different training environments.

Moreover, future studies should employ longitudinal designs that follow the same cohort before and after the clerkship to more accurately measure change. Incorporating objective assessments of knowledge and procedural skills would strengthen the evidence base. Additionally, research should explore whether technology‑enhanced or AI‑supported learning tools, such as adaptive modules, virtual simulation, or automated skills‑tracking, can augment short rotations by providing additional opportunities for deliberate practice and reflection. Such tools may help overcome time constraints and support more robust experiential learning cycles.

## Conclusions

This cross-sectional study compared perceptions, learning preferences, and career interest between medical students surveyed before and after a structured one-week mandatory anesthesiology clerkship. Post-clerkship students more frequently reported anesthesia-related knowledge gains and procedural confidence, particularly in airway management and intravenous access, than pre-clerkship students, suggesting that a well-structured one-week experience is associated with foundational learning gains.

However, the clerkship did not produce statistically significant between-group differences in attitudes toward anesthesiology, and only 16.7% of post-clerkship students reported increased career interest. Rather than treating this null finding merely as a negative result, it should be understood as a positive contribution to the evidence base on minimum effective exposure thresholds. The data indicate that one week represents a floor for foundational learning gains but falls short of the duration required to produce attitudinal or career-related change, a distinction that is directly actionable for curriculum designers. A notable and replicable finding is the shift in learning preferences: students who completed the rotation preferred hands-on workshops (42.6%) over shadowing (31.5%), reversing pre-rotation preferences. This shift has immediate implications for how one-week clerkships should be structured to maximize educational yield within time constraints. These findings establish that short anesthesiology clerkships provide meaningful introductory value but do not suffice to influence specialty choice. Longer rotations, earlier exposure, or longitudinally integrated models remain necessary to achieve attitudinal and career-related impact. Future research employing longitudinal designs with objective outcome measures and exploring whether technology-enhanced or AI-supported tools can augment limited clinical exposure will help determine how to maximize the educational value of constrained curricular time in the perioperative sciences.

## Figures and Tables

**Figure 1:**
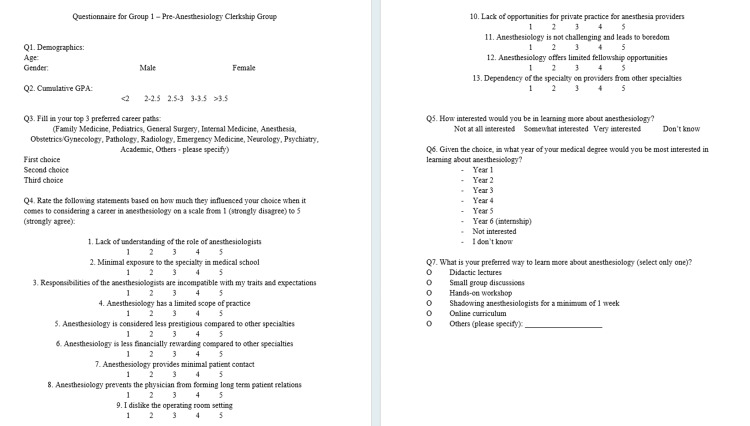
Appendix: questionnaire for group 1: pre-anesthesiology clerkship group.
